# Genetic differentiation and spatiotemporal history of diploidy and tetraploidy of *Clintonia udensis*

**DOI:** 10.1002/ece3.3510

**Published:** 2017-10-25

**Authors:** Juan He, Shengnan Wang, Jia Li, Zelu Fan, Xin Liu, Yiling Wang

**Affiliations:** ^1^ College of Life Science Shanxi Normal University Linfen China; ^2^ College of Animal Science and Technology Nanjing Agricultural University Nanjing China

**Keywords:** *Clintonia udensis*, genetic differentiation, karyotypic characteristics, simple sequence repeat or microsatellites

## Abstract

Polyploidy is an important factor shaping the geographic range of a species. *Clintonia udensis* (*Clintonia*) is a primary perennial herb widely distributed in China with two karyotypic characteristics—diploid and tetraploid and thereby used to understand the ploidy and distribution. This study unraveled the patterns of genetic variation and spatiotemporal history among the cytotypes of *C. udensis* using simple sequence repeat or microsatellites. The results showed that the diploids and tetraploids showed the medium level of genetic differentiation; tetraploid was slightly lower than diploid in genetic diversity; recurrent polyploidization seems to have opened new possibilities for the local genotype; the spatiotemporal history of *C. udensis* allows tracing the interplay of polyploidy evolution; isolated and different ecological surroundings could act as evolutionary capacitors, preserve distinct karyological, and genetic diversity. The approaches of integrating genetic differentiation and spatiotemporal history of diploidy and tetraploidy of *Clintonia udens* would possibly provide a powerful way to understand the ploidy and plant distribution and undertaken in similar studies in other plant species simultaneously contained the diploid and tetraploid.

## 1 INTRODUCTION

Polyploidy is considered important to shape the geographic range of a species. For instance, *Centaurea maculosa* (Asteraceae) of diploid is even more pronounced in the introduced North American range than tetraploid cytotypes (Treier, Broennimann, Normand, Guisan, & Schaffner, 2009); the distribution of diploid and tetraploid races of *Brachypodium distachyon* (Poaceae) is geographically structured with an aridity gradient (Manzaneda, Rey, Bastida, & Weiss‐Lehman, 2012a,b); *B. distachyon* (Poaceae) of tetraploids likely is coresponsible for its occurrence in more arid regions compared with the diploid cytotype (Manzaneda et al., 2012a,b). However, few studies were to understand the ploidy and plant distribution in plant species simultaneously contained the diploid and tetraploid.

The majority of polyploid taxa are of multiple and spatially and/or temporally recurrent origin potentially increasing the polyploid's genomic diversity: The spatiotemporal history of *Knautia arvensis* allows tracing the interplay of polyploid evolution and ecological divergence on serpentine, resulting in a complex evolutionary pattern (Filip et al., 2012); allopolyploid speciation in action with the origins and evolution of *Senecio cambrensis* (Hegarty, Abbott, & Hiscock, 2012); the early stages of polyploidy of rapid and repeated evolution in Tragopogon upon genetic diversity (Soltis, Buggs, Barbazuk, Chamala, & Chester, 2012; Soltis, Buggs, Barbazuk, Schnable, & Soltis, 2009; Soltis & Soltis, 1999); the promiscuous and the chaste of frequent allopolyploid speciation and its genomic consequences in American daisies (Weiss‐Schneeweiss et al., 2012). The geographic distance and the multilocus genetic distance between individuals were computed to evaluate spatial genetic structure of individuals using spatial autocorrelation coefficient; for example, EST‐SSRs were used to characterize polymorphism among 29 Chrysanthemum and *Ajania* spp. accessions of various ploidy levels (Wang, Qi, Gao, & Wang, 2014); genetic variation in polyploid forage grass was assessed the molecular genetic variability in the Paspalum genus (Fernand, Bianca, & Francisco, 2013); public cotton SSR libraries (17,343 markers) were curated for sequence redundancy using 90% as a similarity cut‐off (Anna, David, Jean, & Olivier, 2012); spatiotemporal history of the diploid–tetraploid complex of *K. arvensis* (Dipsacaceae) upon evolution on serpentine and polyploidy (Filip et al., 2012), genetic, and genomic attributes in the success of polyploids (Pamela & Douglas, 2000). Species with little genetic variability may suffer from reduced fitness and may not show the potential evolutionary necessary under the changed environment (Bodare, Tsuda, Ravikanth, Shaanker, & Lascoux, 2013; Gong, Zhan, & Wang, 2015). Genetic diversity and differentiation under different levels could be assessed to provide the understanding of the evolutionary history within species. Numbers of PCR‐based techniques including Simple Sequence Repeat or microsatellites (SSR) were used to analysis the polymorphisms of genetic diversity and spatiotemporal history: genetic analysis and molecular characterization of Chinese sesame (*Sesamum indicum* L.) cultivars using SSR markers (Wu, Yang, Liu, Tao, & Zhao, 2014); genetic diversity and population structure assessed by SSR marker in a large germplasm collection of grape (Francesco et al., 2013); genetic diversity, genetic structure, and demographic history of *Cycas simplicipinna* (Cycadaceae) assessed by SSR markers (Feng, Wang, & Gong, 2014).

**Figure 1:**
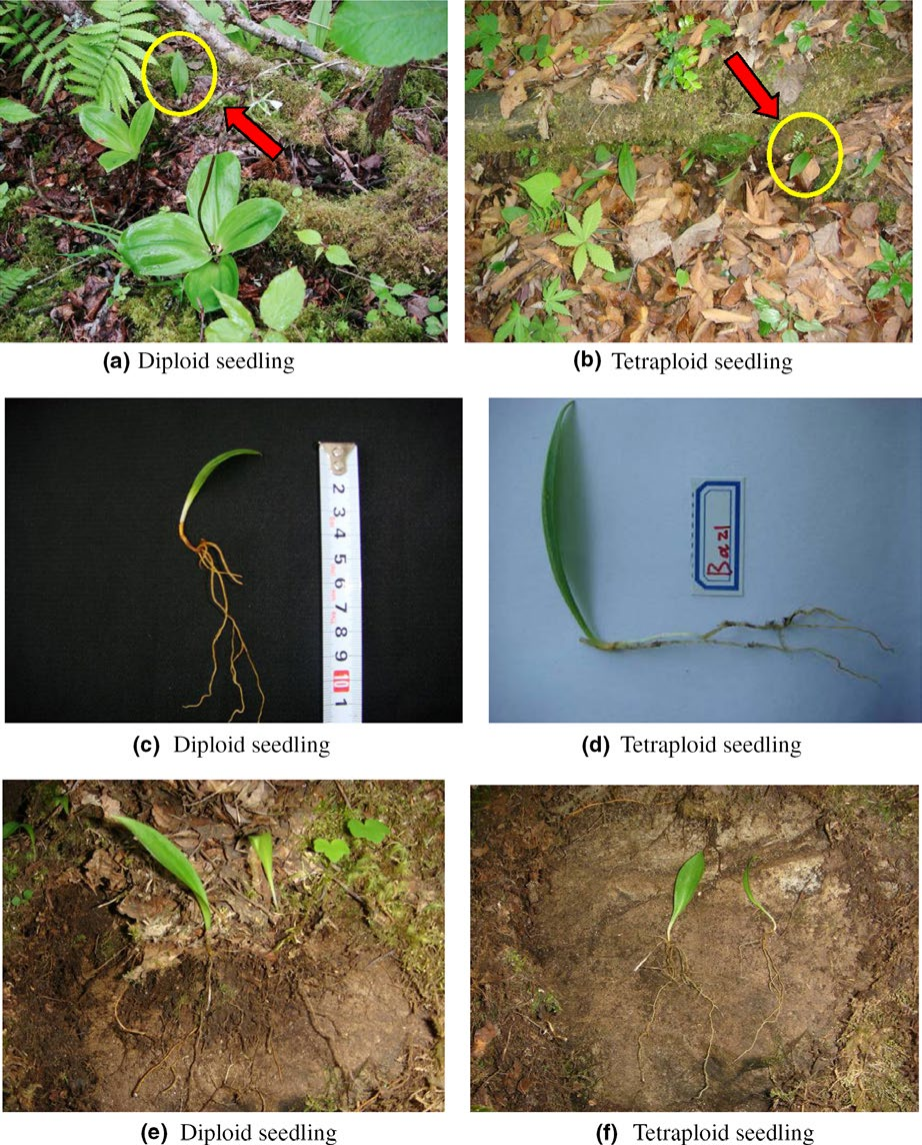
(a, c, e) are seedling of *Clintonia udensis* (2n); (b, d, f) are seedling of *C. udensis* (4n). The red arrow means the seedling of (2n) in picture a and the seedling of *C. udensis* (4n) in picture b; picture c and d means the seeding size of *C. udensis* (2n and 4n), picture e and f means the root of *C. udensis*

*Clintonia udensis* (Liliaceae)Trautv. et Mey. (*Clintonia*) is perennial with globose or ellipsoid blackish‐blue berry widely distributed in the area of Japano‐Himalayan element from the Japanese Islands to the Himalayan Mountains in northeastern Asia at 1,600 m to 4,000 m above sea level (Kanai, 1963; Kim, Kim, & Chase, 2016; Wagner, 1973; Wang et al., 1978). The diploid of *C. udensis* (2n = 14 and 4n = 28) was distributed in northwest Yunnan of China and Primorskiy Kray in Russia while the tetraploid were spread Yunnan, the Himalayas, Japan, and Mount Hualongshan (Figure 1) (Li, Chang, & Yuan, 1996; Wang et al., 1978). Those areas were studied for the genetic diversity and phylogeography, indicate that the history of *C. udensis* involved both long‐distance migration and the tectonic events of Mountains in East Asia; and mixed‐mating—breeding system, limited gene flow, environmental stress, and historical factors may be the main factors causing geographic differentiation in the genetic structure of *C. udensis* (Wang, Guo, & Zhao, 2011; Wang, Li, Guo, Li, & Zhao, 2010). However, only two regions (Hunan and Shaanxi provinces) of the *C. udensis* range were simultaneously contained the diploid and tetraploid. Geographically the diploid and tetraploid are parapatric or partially was overlapping (Li & Chang, 1996; Li et al., 1996), the diploid and tetraploid of *C. udensis* was no corresponding morphological differentiation except that seeds of tetraploid are constantly bigger than that of diploid (Li & Chang, 1996; Li et al., 1996), the derivation from lower ploid level to higher ploidy level is an irreversible process (Huang, 1990), and the tetraploid types generally could adapt new different environment than diploid types (Huang, 1990; Li & Chang, 1996; Li et al., 1996). Thus, *C. udensis* was an ideal plant to understand the ploidy and distribution based on diploid and tetraploid. This study was (1) to analyze the genetic level of *C. udensis* in both diploid and tetraploid using the SSR markers, (2) to explain the intraspecific genetic differentiation of *C. udensis* in populations from the diploid and tetraploid types, (3) to describe the phylogeographic population relationships, and to explore the origin of tetaploid by recurrent polyploidization or by colonization. The approaches of integrating genetic differentiation and spatiotemporal history of diploidy and tetraploidy of *Clintonia udens* provide a powerful way to understand the ploidy and plant distribution and could be undertaken in similar studies in other plant species simultaneously contained the diploid and tetraploid.

## 2 MATERIALS AND METHODS

### 2.1 Plant material

The fresh leaves of 31–41 individuals were collected randomly in four natural populations (HLN, JHL, HLB, and MLZ) of *C. udensis*. Among the four populations, the karyotypic characteristics of HLN and JHL populations are diploid cytotype while HLB and MLZ are tetraploid cytotype. The HLN and HLB populations located at Hualongshan Mountains in Shaanxi Province while JHL and MLZ located at Shennongjia Mountains in Hunan province. This study was included total of 152 individuals (Table 1).

**Table 1:** Location between experimental materials and environmental factors

Population	Location	Samples	Longitude	Latitude	Altitude (m)	Isothermality (*100)	Precipitation of warmest quarter (mm)	Precipitation of coldest quarter (mm)
HLB (tetraploid)	Hualongshan Shaanxi	39	31.10	109.68	2,033	27	414	42
HLN (diploid)	Hualongshan Shaanxi	31	32.02	109.58	2,404	27	454	48
JHL (diploid)	Jinhouling Hubei	41	31.78	110.50	2,479	25	505	60
MLZ (tetraploid)	Mulinzi Hubei	41	30.05	110.33	2,042	27	600	72

### 2.2 Microsatellites amplification

The leaves were used to extracted genomic DNA followed the method of CTAB (Doyle & Doyle, 1987). The locus of polyploids is potentially higher than the diploids upon the number of alleles (Pfeiffer, Roschanski, Pannell, Korbecka, & Schnittler, 2011; Teixeira, Rodríguez‐Echeverría, & Nabais, 2014). Polymorphic 20 microsatellite loci were tested from an initial set of putative for developed of Liliaceae. Polymorphic 20 microsatellite loci were tested from an initial set of putative for developed of Liliaceae (Chung & Jack, 2003; Guo, Wang, Li, & Zhao, 2011). The targeted and polymorphism loci were amplified successfully in eleven of these 20 primers used in this study (Table 2). The data sets of SSR markers were collected by polyacrylamide gel (PAGE) silver staining. PCR that did not produce bands or that had different size were repeated.

**Table 2:** The SSR primers used in the study

	Random primer pairs and annealing temperature		Random primer pairs and annealing temperature
ccSSR‐6	F:CGACCAATCCTTCCTAATTCAC	ccSSR‐7	F:CGGGAAGGGCTCGKGCAG
56.2°C	R:AGAAAAGMAAGGATATGGGCTC	56.2°C	R:GTTCGAATCCCTCTCTCTCCTTTT
ccSSR‐9	F:GAGGATACACGCAGARGGARTTG	ccSSR‐12	F:CCAAAAACTTGGAGATCCAACTAC
58°C	R:CCTATTACAGAGATGGTGYGATTT	56.2°C	R:TTCCATAGATTCGATCGTGGTTTA
ccSSR‐15	F:GCTTATGACCTCCCCCTCTATGC	ccSSR‐16	F:TACGAGTCACCCCTTTCATTC
58°C	R:TGCATTACAGACGTATGATCATT	56.2°C	R:CCTGGCCCAACCCTAGACA
ccSSR‐17	F:CACACCAATCCATCCCGAACT	ccSSR‐20	F:CCGCARATATTGGAAAAACWACAA
60°C	R:GGTGCGTTCCGRGGTGTGA	55°C	R:GCTAARCAAATWGCTTCTGCTCC
ccSSR‐21	F:CCACCCCGTCTCSACTGGATCT	ccSSR‐22	F:CCGACCTAGGATAATAAGCYCATG
56.2°C	R:AQAAAATAGCTCGACGCCAGGAT	58°C	R:GGAAGGTGCGGCTGGATC
ccSSR‐23	F:AYGGRGGTGGTGAAGGGAG		
58°C	R:TCAATTCCCGTCGTTCGCC		

### 2.3 Data analysis

The software of GenoDive was used to calculate effective number of alleles per locus (Ae) and heterozygosity within each population (Hs) (Meirmans & Vartienderen, 2004). GenAlex 6.5 was used for further analysis of the multilocus data (Peakall & Smouse, 2012), including number of alleles, genetic diversity, Shannon's information index, finescale genetic structure (Kloda, Dean, Maddren, MacDonald, & Mayes, 2008; Obbard, Harris, & Pannell, 2006; Peakall & Smouse, 2006; Sampson & Byrne, 2012; Teixeira et al., 2014). PhiPT was used to determine the genetic differentiation between populations, and a Mantel test was performed for genetic and geographic distances (Assoumane, Zoubeirou, Rodier‐Goud, & Favreau, 2012; Mantel, 1967). The genetic similarity between samples was explored for principal component analysis (PCA) using PolySat, an R package for polyploid microsatellite analysis in ecological genetics (Clark & Jasieniuk, 2011). The STRUCTURE software version 2.2 was used to assess the population genetic structure following a model‐based Bayesian assignment that allows mixed ancestry of individuals (admixture model). (Evanno & Regnaut, 2005; Hubisz, Falush, Stephens, & Pritchard, 2009; Pritchard, Stephens, & Donnelly, 2000), and the genetic clusters (*K*) of individuals were assumed in Hardy–Weinberg and linkage equilibria were given number from *K *=* *1 to *K *=* *4 to investigate under the correlated allele frequencies model by running 100,000 iterations. A Neighbor‐joining (NJ) tree was performed the genetic relationships among populations ((Nei, Tajima, & Tateno, 1983; Rambaut, 2014).

## 3 RESULTS

### 3.1 Genetic diversity

All amplified loci were highly polymorphic and displayed up to 4.15 alleles per sample from the 12 microsatellite loci of total of 50 alleles, and the number of private alleles and different alleles of diploid were generally higher than tetraploid within each population (An) (Wilcoxon test, *p* = .01). The average number of effective alleles per locus and high values of heterozygosity was similar in all populations. Significantly higher values were also found in all populations for the Shannon's information index (I) (Table 3, Wilcoxon tests, *p* = .035). Two cytotyped were significant differences found in these diversity estimators (Wilcoxon test, *p* = .046) (Table 3).

**Table 3:** Diversity of the studied populations of *Clinton udensis* obtained from the analysis of microsatellite loci

Population	*N*	An	Ae′	PA	Hs	I	H
JHL (diploid)	39	0.719	1.291	12	0.156	0.222	0.085
HLN (diploid)	31	0.438	1.191	7	0.101	0.143	0.131
Total diploid	70	0.578	1.241	19	0.129	0.182	0.108
HLB (tetraploid)	41	0.313	1.112	4	0.065	0.094	0.058
MLZ (tetraploid)	41	0.531	1.216	8	0.114	0.162	0.098
Total tetraploid	82	0.422	1.164	12	0.090	0.128	0.078
Total species	152	0.500	1.202		0.109	0.155	0.193

*N*, number of individuals; An, number of alleles in each population; Ae′, average number of effective alleles per locus; PA, number of private alleles per population; Hs, heterozygosity within populations; I, Shannon's Information Index; H, genetic diversity.

Twenty‐nine alleles of the observed 50 alleles were shared by all populations while the rest were exclusively found in diploid or tetraploid population. About 38% of private alleles were found only in diploid populations while 24% were found exclusively in the tetraploid populations, and JHL was the highest while HLB was the lowest private alleles. And the locus 4 was the locus with the highest number of private alleles.

### 3.2 Population differentiation and genetic structure

PhiPT distances were ranged between 0.673 (HLN/HLB) and 0.813 (JHL/MLZ) and were statistically significant between all studied populations (Table 4). It indicated that the genetic diversity among populations were occurred with 77% while within populations were contributed 23% (Table 5). Whether diploid (80%, *p *=* *.001) or tetraploid (69%, *p *=* *.001) populations, the genetic variability was also both mainly found among populations.

**Table 4:** Pairwise PhiPT genetic distance between the studied populations

Population	JHL (diploid)	HLN (diploid)	HLB (tetraploid)	MLZ (tetraploid)
JHL (diploid)	0	0.001	0.001	0.001
HLN (diploid)	0.801	0	0.001	0.001
HLB (tetraploid)	0.767	0.673	0	0.001
MLZ (tetraploid)	0.813	0.768	0.693	0

PhiPT values are shown below diagonal. Probability based on 1,000 permutations was shown above diagonal.

**Table 5:** Analysis of molecular variance (AMOVA) showing the partitioning of genetic variation within and between populations of *Clinton udensis*

Source of variation	*df*	SS	MS	Est. Var.	%	*p*
Among diploid populations	1	206.623	206.623	5.939	80	.001
Within diploid populations	68	100.491	1.478	1.478	20	
Among tetraploid populations	1	125.659	125.659	3.032	69	.001
Within tetraploid populations	80	107.463	1.343	1.343	31	
Among populations	3	524.322	174.774	4.580	77	.001
Within populations	148	207.955	1.405	1.405	23	

**Figure 2:**
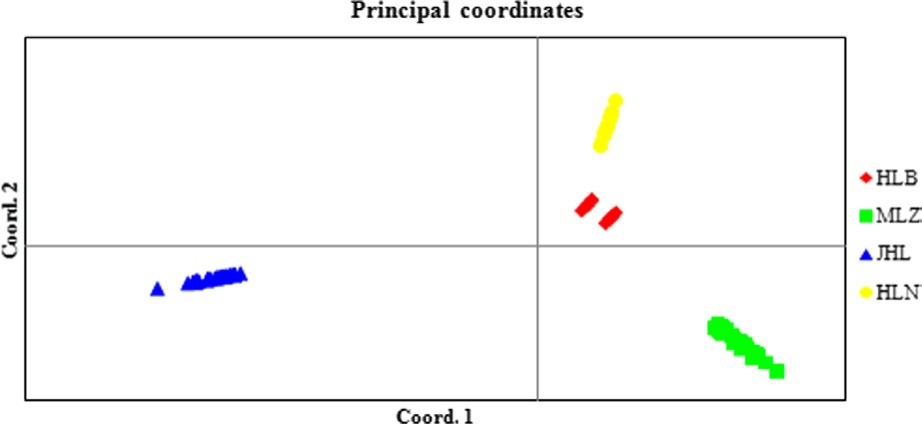
Principal component analysis (PCoA) of the 152 individuals based on microsatellite loci. HLN and JHL: diploid population of *Clintonia udensis*; HLB and MLZ: tetraploid population of *C. udensis*. The abbreviation of HLN means Hualongshan, JHL means Jinhouling, HLB means Hualongshan, and MLZ means Mulinzi

**Figure 3:**
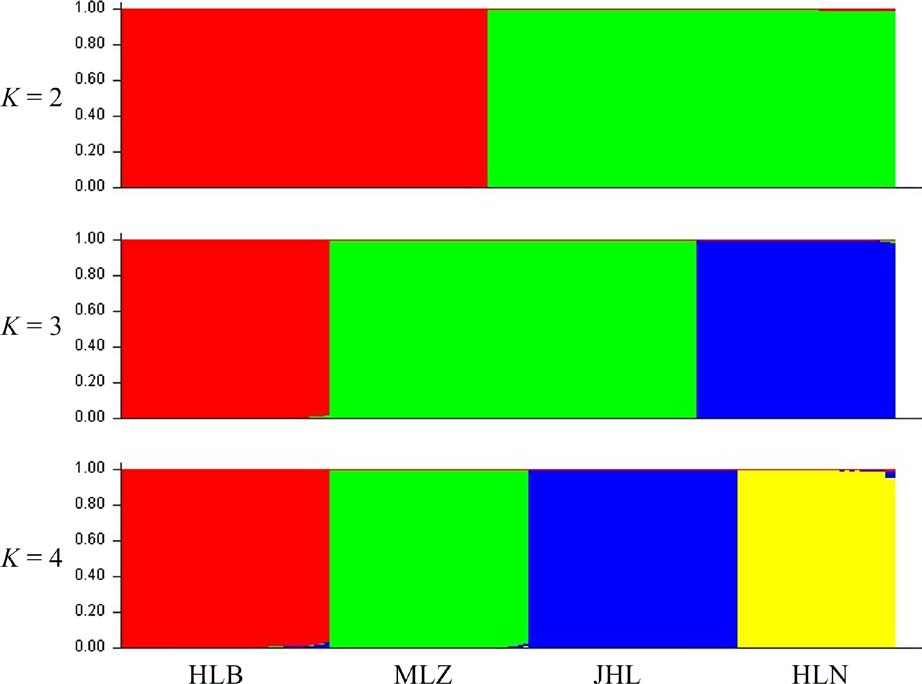
Means of assignment coefficients to each group (*K*) per population. HLNand JHL: diploid population of *Clintonia udensis*; HLB and MLZ: tetraploid population of *C. udensis*. The abbreviation of HLN means Hualongshan, JHL means Jinhouling, HLB means Hualongshan, and MLZ means Mulinzi

The samples were separated into three groups by the PCA, with some overlap (Figure 2). The correlation of genetic and geographic distances was significant revealed under the Mantel test based on Euclidean distances (*R* ^2^ = 0.024, *p *=* *.001). Bayesian analysis showed populations of diploid JHL, and HLN were grouped into one cluster while populations of tetraploid MLZ and HLB were grouped into the other cluster that with the value of *K *=* *2, which was dominant in each of the two cytotype; the population of diploid HLN and population of tetraploid HLB solely gathered a cluster, respectively, with the value of *K *=* *3; four populations were, respectively, gathered different cluster and were easily distinguished with the value of *K *=* *4 (Figure 3).

**Figure 4:**
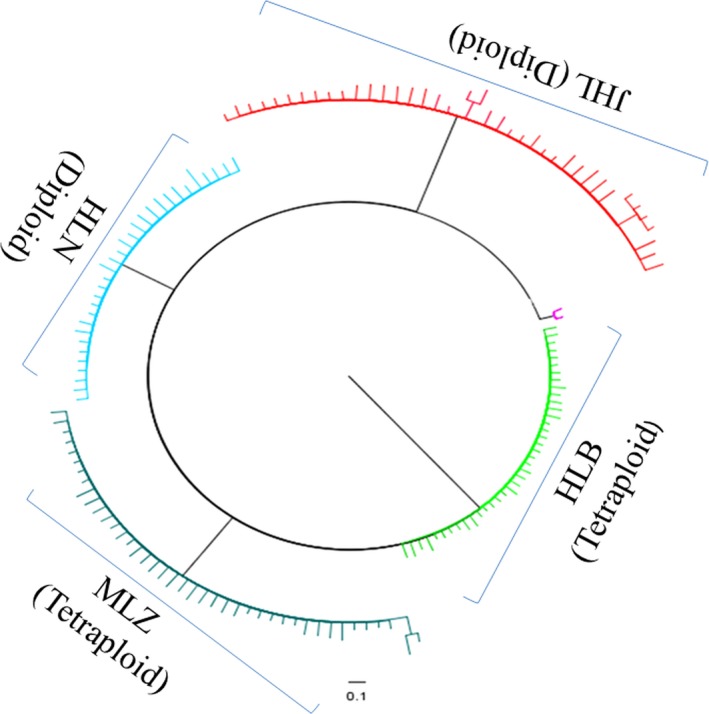
Dendrogram of *Clintonia udensis* samples based on genetic distance. HLN and JHL: diploid population of *C. udensis*; HLB and MLZ: tetraploid population of *C. udensis*. The abbreviation of HLN means Hualongshan, JHL means Jinhouling, HLB means Hualongshan, and MLZ means Mulinzi

The dendrogram (Figure 4) obtained with the Neighbor‐joining clustering method showed that the studied populations were separated into three clades with high bootstrap values. It is the same as STRUCTURE and PCA.

## 4 DISCUSSION

It is hypothesized that polyploids were contributed to greater genetic and biochemical diversity, and thus, polyploids are expected to have larger geographic ranges and/or occur in more habitats than diploids. The analysis of four populations of *C. udensis* using 11 polymorphic SSR revealed clear separation between the diploid and tetraploid populations and moderate values of genetic diversity. The variability was accounted for 67% in diploid and tetraploid while variation was accounted for 77% of the total genetic diversity.

The genetic differentiation of diploid and tetraploid populations was probably contributed to the geographic barrier. PCA *C. udensis* populations were more diverse in diploid than in tetraploid in terms of higher genetic diversity and isolation by distance also indicated in the Mental test, the colonization history of *C. udensis* was possibly due to the medium genetic diversity in tetraploid upon *C. udensis* originated in East Asian and then through the Beringia bridge spread to North American, similar studies were reported in alfalfa or other herbs (Li & Chang, 1996; Qiang et al., 2015). Our study indicates bottleneck effect was contributed to possible loss of genetic diversity during migration and environmental filters arised in North American or genetic drift, isolated evolution of the migrant genotypes was probably lead to the current genetic differentiation between diploid and tetraploid.

Diploid ancestors were probably arise during the middle of the Tertiary period to be restricted to refugia by expanding forest vegetation (Li & Chang, 1996; Wang et al., 2010). Mechanisms of allopatric differentiation taken place upon spatial isolation and population size fluctuations were leaded to the genetic differentiation observed in the diploid populations, illustrated by the significance of refugia for preserving rare and distinct genetic diversity of diploid within *C. udensis*. Two factors could also be contributed to the medium genetic diversity of *C. udensis* tetraploid: Local and glacial survival might be leaded to tetraploid with a medium genetically diverse; the lack of genetic bottlenecks and the maintenance of large effective population sizes was contributed to genetic diversity of tetraploid population during postglacial migration.

Marked diversity of polyploidy complexes showed frequent component of polyploid evolution for recurrent origin exhibited striking differences in morphology, ecology, or genetic profiles formed polyploid lineages (Brochmann, Soltis, & Soltis, 1992; Segraves, Thompson, Soltis, & Soltis, 1999; Soltis & Soltis, 1999; Soltis, Soltis, Pires, & Kovarik, 2004; Soltis, Soltis, & Tate, 2003). Diploids and tetraploids of *C. udensis* observed in China for its mosaic pattern while the contact zones found no populations with mixed ploidal levels respected to the origin of the tetraploids, either be recent autopolyploidization event or the secondary contact of formerly allopatric populations (Schmickl, Paule, Klein, Marhold, & Koch, 2012). Moreover, the higher number of private alleles of diploid population might reflect the existence of glacial refugia or higher historical or contemporary gene flow contribute to diploid and tetraploid of population (Li & Chang, 1996).

The tetraploids of *C. udensis* were formed independently by autopolyploidization (Li & Chang, 1996; Li et al., 1996). The local diploid and tetraploids suggested the basis of phenotypic similarities and habitat preferences (Kaplan, 1998). The strong introgression of the tetraploid genotype into the diploids ruled out was due to the virtual lack of triploid hybrids (Kolář, Fér, Štech, Trávníček, & Dušková, 2012; Kolář, Štech, Trávníček, Rauchová, & Urfus, 2009). In addition, no indication of across‐ploidy genetic admixture was in the other contact zone between the tetraploids and diploids, two individuals of the tetraploid population HLB single gathered a cluster indicated the complex mechanism of tetraploid of *C.  udensis*.

In summary, polyploidy is contributed to shape the geographic range of a species. Two regions (Hunan and Shaanxi provinces) distributed the diploid (HLN, JHL) and tetraploid (HLB and MLZ) of *C. udensis* (2n = 14 and 4n = 28) were arised from the isolated and different ecological surroundings, which could be evolutionary capacitors to preserved distinct karyological, and diploid–tetraploid complex that exhibits an intriguing pattern of eco‐geographic differentiation. However, polyploidy is a significant and complex mode of species formation in plants, therefore, firstly, solely depend on the genome DNA of SSR markers of plant distribution and polyploidy was limited and Reduced‐Representation Genome Sequencing should be used to the polyploidy genome analysis of phylogeography to confirm the relationship of the ploidy and plant distribution in plant species simultaneously contained the diploid and tetraploid in the future; secondly, only four populations simultaneously contained the diploid and tetraploid are difficult to explain the factors involved in the origin and establishment of polyploids in nature, and the rest samples of other regions of YLXS (*C. udensis*, 4n = 28) and DGK, HSK, LW, MLG, GBS, LTDZ, HHL, TBS, FP, KZQ, EMS, JYH (*C. udensis*, 2n = 14) should be collected and analyzed in the future study.

## CONFLICT OF INTEREST

None declared.

## AUTHOR CONTRIBUTION

JH, SNW, and YLW designed the study; JL and ZLF performed data management; JH and SNW analyzed the data and interpreted the results; JH, SNW, and YLW wrote the manuscript.
